# Dolutegravir Has No Effect on the Pharmacokinetics of Oral Contraceptives With Norgestimate and Ethinyl Estradiol

**DOI:** 10.1177/1060028015580637

**Published:** 2015-07

**Authors:** Ivy H. Song, Julie Borland, Shuguang Chen, Toshihiro Wajima, Amanda F. Peppercorn, Stephen C. Piscitelli

**Affiliations:** 1 GlaxoSmithKline, Research Triangle Park, NC, USA; 2 Shionogi & Co, Ltd, Osaka, Japan

**Keywords:** integrase inhibitor, HIV, drug interaction, healthy subjects, pharmacokinetics

## Abstract

**Background:** Dolutegravir (DTG; Tivicay; ViiV Healthcare, Research Triangle Park, NC) is an HIV-1-unboosted integrase inhibitor with no cytochrome P450 or uridine 5′diphosphate-glucuronosyltransferase inhibition or induction. As DTG is administered to HIV-1–infected women receiving oral contraceptives, assessing the potential for drug interactions was warranted. **Objective:** To determine the impact of DTG on the pharmacokinetics (PK) and pharmacodynamics (PD) of a common oral contraceptive, norgestimate/ethinyl estradiol (NGM/EE; Ortho-Cyclen; Ortho-McNeil-Janssen Pharmaceuticals, Inc, Raritan, NJ). **Methods:** This randomized, 2-period, double-blind, placebo-controlled study was conducted within 1 menstrual cycle at 1 clinical center in the United States; 16 women were enrolled. Participants received NGM 0.25 mg/EE 0.035 mg throughout the study. During days 1 to 10, they were randomized to receive twice-daily DTG 50 mg or matching placebo with food and switched to the other treatment during days 12 to 21. **Results:** Ratios of area under the concentration-time curve from time 0 until end of the dosage interval (AUC_0-τ_), maximum plasma concentration, and concentration at the end of the dosage interval of norelgestromin with DTG treatment to the same PK parameters with placebo treatment were 0.975, 0.890, and 0.932, respectively; for EE, ratios were 1.03, 0.99, and 1.02, respectively. No significant differences in luteinizing hormone, follicle-stimulating hormone, and progesterone were detected on days 1, 10, 11, 21, and 22. DTG steady-state AUC_0-τ_ was similar to historical data. No severe or grade 3/4 adverse events occurred. **Conclusions:** DTG had no effect on NGM/EE PK or PD. NGM/EE can be administered with DTG without dose adjustment.

## Introduction

Dolutegravir (DTG; Tivicay; ViiV Healthcare, Research Triangle Park, NC) is an HIV-1 integrase strand transfer inhibitor approved in the United States for once-daily or twice-daily dosing without pharmacokinetic (PK) boosters.^1^ DTG is metabolized primarily by uridine 5′-diphosphate-glucuronosyltransferase (UGT) with cytochrome P450 (CYP) playing a minor role.^2^ However, DTG displays no significant CYP or UGT inhibition or induction^2^ and does not require dose adjustments of other drugs that are metabolized by CYP3A, such as midazolam and methadone.^3,4^ DTG is an inhibitor of renal transporters, including organic cation transporter 2 (OCT2), and has the potential to increase the concentration of drugs that are OCT2 substrates, for example, metformin.

Norgestimate/ethinyl estradiol (NGM/EE; Ortho-Cyclen; Ortho-McNeil-Janssen Pharmaceuticals, Inc, Raritan, NJ) is a widely prescribed combination oral contraceptive, containing both a synthetic estrogen and progesterone,^5^ administered as a fixed dose of NGM 0.25 mg and EE 0.035 mg throughout the 21-day dosing cycle, with an additional 7 placebo tablets to maintain a 28-day cycle. Oral contraceptive components are primarily metabolized through the oxidative, reductive, and conjugative pathways. NGM undergoes both oxidative and reductive metabolism. CYP is involved in the oxidative metabolism of EE,^6^ and the induction of CYP has been shown to increase the clearance of oral contraceptives, thus decreasing their plasma concentration.^7,8^ Clinical evaluations of drug interactions between antiretroviral drugs showed that concentrations of oral contraceptives may be reduced by ritonavir-boosted protease inhibitors, cobicistat-boosted elvitegravir, efavirenz, and nevirapine, and alternative methods of contraception should be used.^9^ Antiretrovirals that do not interact with oral contraceptives provide greater treatment flexibility.

Because DTG is administered to HIV-1–infected women receiving oral contraceptives, a study to assess the potential for a drug interaction was warranted. Although the observed lack of induction of CYP enzymes by DTG^2^ suggests that the possibility of a drug interaction is low, this study was performed to formally determine the impact of DTG on the PK (primary) and pharmacodynamics (PD, secondary) of NGM/EE in a controlled clinical pharmacology study. Because oral contraceptives are not known to induce or inhibit UGT1A1 or CYP3A4, the study was primarily designed to examine the effect of DTG on NGM/EE and not vice versa.

## Materials and Methods

### Study Design

This was a randomized, single-center, 2-period, double-blind, placebo-controlled, crossover study in healthy HIV-negative women, conducted within 1 menstrual cycle. Written informed consent was obtained from each participant before the performance of any study-specific procedures. The study was conducted in accordance with the International Conference on Harmonisation of Technical Requirements for Registration of Pharmaceuticals for Human Use Good Clinical Practice, all applicable subject privacy requirements, and the ethical principles that are outlined in the 2008 Declaration of Helsinki. The study was approved by the institutional review board of the study site (Independent Investigational Review Board, Plantation, FL).

Healthy women, 18 to 40 years old, with body mass index 19 to 30 kg/m^2^, body weight ≥50 kg and <114 kg, and alanine aminotransferase, alkaline phosphatase, and bilirubin levels ≤1.5 times the upper limit of normal were eligible. Women of childbearing potential were required to use NGM/EE in combination with 1 of the following contraceptive methods: complete abstinence from intercourse for at least 14 days before the first dose of study medication until after study monitoring, a barrier method plus a spermicide, or sterilization of male partner. Those positive for hepatitis B or C within 3 months of screening, those with current or chronic history of liver disease, and those with positive prestudy drug/alcohol or HIV test results as well as those who were pregnant or lactating, unwilling or unable to follow the protocol, and not using appropriate methods of contraception or abstaining from tobacco were excluded from the study. Women who had conditions or who were taking concurrent medications that would adversely affect hormone levels were also excluded.

**Figure 1.**
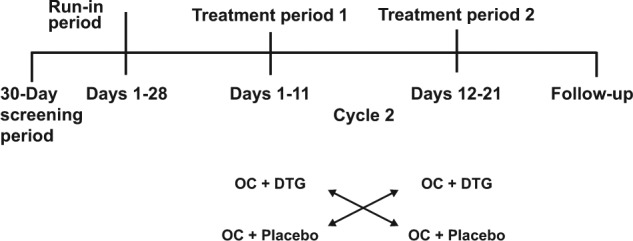
Study design: Those who were already stable on NGM/EE were permitted to skip the run-in period and proceed to the treatment periods. Abbreviations: DTG, dolutegravir; NGM/EE, norgestimate/ethinyl estradiol; OC, organic cation.

The study consisted of a screening phase (within 30 days before day 1, a treatment phase (2 treatment periods ± a prior run-in period), and a follow-up phase scheduled approximately 7 to 14 days after the last dose of study drug (Figure 1). Those who were not already on a stable regimen of NGM/EE received a run-in period over 1 cycle before the study treatments in which they started NGM/EE dosing near the beginning of the first day of a menstrual cycle for 21 days to evaluate tolerability and regulate hormone secretion, followed by a washout period of 7 days.

Participants received NGM 0.25 mg + EE 0.035 mg throughout the study. During treatment period 1, they were randomly assigned to receive DTG 50 mg twice daily or placebo with a moderate-fat (~30% fat) meal every 12 hours for 10 days followed by NGM/EE only on day 11. The purpose of dosing with food was to maximize the potential for a drug-drug interaction with DTG because DTG exposure increased with the coadministration with food. During treatment period 2 (days 12 to 21), those who had taken DTG were switched to placebo, and those who had taken placebo were switched to DTG (Figure 1). All participants returned to the study center 7 to 14 days after the last dose of study drug for follow-up evaluations, including physical examination, vital sign measurements, hematology and clinical chemistry assessments, and serum pregnancy tests.

### Pharmacokinetic Assessments

Serial blood samples for PK analysis were collected at the following time points relative to the proposed time of dosing on days 10 and 21: predose, 0.5, 1, 1.5, 2, 2.5, 3, 4, 6, 8, 10, 12, and 24 hours postdose for norelgestromin (NGMN; the active metabolite of NGM) and EE, and predose, 1, 2, 3, 4, 6, 8, and 12 hours postdose for DTG. Predose samples were also collected on days 8, 9, 19, and 20 for NGM and EE. Bioanalysis of plasma samples for DTG was performed by QPS (Newark, DE) with a validated analytical method based on protein precipitation followed by high-performance liquid chromatography-tandem mass spectrometry (HPLC-MS/MS) analysis.^10^ Bioanalysis of the plasma samples for NGMN and EE was performed by PPD Inc (Richmond, VA) with validated analytical methods. The NGMN method was based on liquid-liquid extraction followed by HPLC-MS/MS analysis. The lower limit of quantification for NGMN was 0.02 ng/mL with a higher limit of quantification of 10.0 ng/mL. The EE method was based on extraction with an organic solvent, followed by derivatization and then HPLC-MS/MS analysis. The lower limit of quantification for EE was 2.00 pg/mL, with a higher limit of quantification of 500 pg/mL.

Noncompartmental PK analyses using WinNonlin, version 5.3 (Pharsight, Cary, NC) were performed to determine parameters for DTG, NGMN, and EE, including area under the concentration-time curve from time 0 until the end of the dosage interval (AUC_0-τ_) using the combination of linear-up and log-down trapezoidal rules, maximum concentration (*C*_max_), minimum concentration (*C*_min_). Actual elapsed time from dosing was used in the derivation of all PK parameters.

### Pharmacodynamic Assessments

Plasma analysis for luteinizing hormone (LH), follicle-stimulating hormone (FSH), and progesterone was performed at screening and on days 1, 10, 11, 21, and 22. For each biomarker (LH, FSH, and progesterone), the profiles for both treatment sequences (treatment sequences AB and BA) were plotted using box plots displaying the median, range, and 25th and 75th percentiles. Because of large variability and small sample size, formal statistical comparison of LH, FSH, and progesterone by treatment was not performed.

### Safety Assessments

Adverse event (AE) and serious AE (SAE) data were collected and recorded from the start of study treatment until the follow-up 7 to 14 days after the last dose. Any abnormal laboratory assessments that were considered clinically significant were recorded as AEs or SAEs if they met the respective definitions. All clinically significant changes from baseline were followed until resolution or stabilization, as determined by the investigator or his/her designee.

### Statistical Analyses

A sample size of 16 to obtain 14 evaluable participants was chosen, assuming a within-subject variability of 15% and a true ratio of 1, which would provide >90% power to demonstrate lack of interaction within the bioequivalence limit of 0.80 to 1.25.

Geometric least squares (GLS) mean ratios and 90% CIs were generated by the mixed-effect model, with treatment and period as fixed effects and subject as a random effect for within-subject treatment comparison. A *P* value <0.05 for period effect was considered to be statistically significant. To assess the lack of effect of multiple doses of DTG on the PK (AUC_0-τ_) of NGMN and EE, a bioequivalence approach was followed, using Schuirmann’s two 1-sided *t* test procedure with α = 0.05 for each test.^11^ Lack of effect was tested by estimating the ratio of the GLS means for AUC_0-τ_ of each of NGMN and EE in the presence of DTG to the GLS mean of each of NGMN and EE alone; a ratio of <0.8 or >1.25 suggested a drug interaction. All statistical analyses were performed using SAS version 9.1.

## Results

### Patient Demographics and Disposition

Overall, 16 healthy women (15 white and 1 African American) were enrolled, and 15 (94%) completed the study; 12 individuals were required to receive a 1-cycle run-in of NGM/EE. One woman withdrew for personal reasons unrelated to the study. Mean (SD) values were 31.1 (7.5) years for age, 24.7 (3.0) kg/m^2^ for body mass index, and 64.5 (9.5) kg for weight. All participants were of Hispanic ethnicity, and the majority (94%) were of European heritage.

### Pharmacokinetics

**Figure 2.**
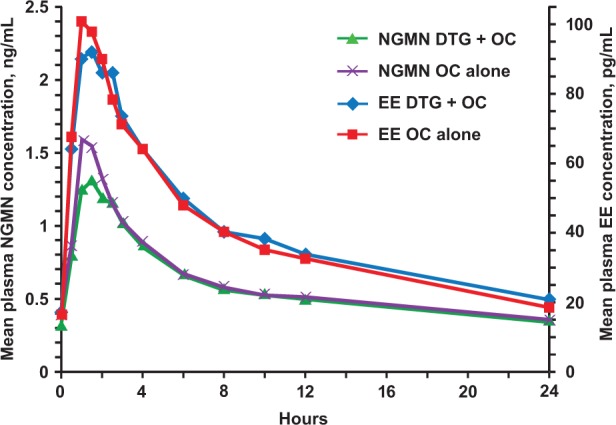
Mean concentration-time profiles of EE and NGMN with and without DTG. Abbreviations: DTG, dolutegravir; EE, ethinyl estradiol; NGMN, norelgestromin; OC, organic cation.

Plasma concentrations of NGMN and EE are shown in Figure 2, and PK parameters are summarized in Table 1.

**Table 1.** Pharmacokinetic NGMN and EE Parameters.

Parameters, Geometric Mean (CV%)^a^	NGM/EE Once Daily + DTG 50 mg bid, n = 16	NGM/EE Once Daily Alone, n = 16	Geometric Least-Squares Mean (90% CI)^b^
NMGN			
AUC_0-τ_, ng·h/mL	13.8 (16)	14.1 (25)	0.98 (0.91, 1.04)
*C*_max_, ng/mL	1.4 (18)	1.6 (30)	0.89 (0.82, 0.97)
*C*_min_, ng/mL	0.3 (26)	0.3 (30)	0.93 (0.85, 1.03)
EE			
AUC_0-τ_, pg·h/mL	952 (19)	916 (27)	1.03 (0.96, 1.11)
*C*_max_, pg/mL	100 (22)	101 (25)	0.99 (0.91, 1.08)
*C*_min_, pg/mL	16.6 (19)	16.4 (33)	1.02 (0.93, 1.11)

Abbreviations: AUC_0-τ_, area under the concentration-time curve over the dosing interval; *C*_max_, maximum observed concentration; *C*_min_, minimum observed concentration; CV, coefficient of variation; DTG, dolutegravir; EE, ethinyl estradiol; NGM/EE, norgestimate/ethinyl estradiol; NMGN, norelgestromin.

a Values are CV% unless otherwise noted.

b Ratio represents pharmacokinetic parameters from treatment with NGM/EE + DTG versus NGM/EE alone.

Ratios comparing NGMN PK parameters with or without DTG treatment ranged from 0.89 to 0.98; corresponding ratios comparing EE PK parameters ranged from 0.99 to 1.03 (Table 1). All 90% CIs for the ratios were within the predetermined range of 0.8 to 1.25, which defined lack of interaction. There was no statistically significant period effect on any PK parameters for either plasma NGMN or EE (*P* > 0.05). The geometric mean of AUC_0-τ_ for DTG was 68.6 µg·h/mL, and the mean *C*_max_ and *C*_min_ were 7.8 and 3.8 µg/mL, respectively.

### Pharmacodynamics

**Figure 3.**
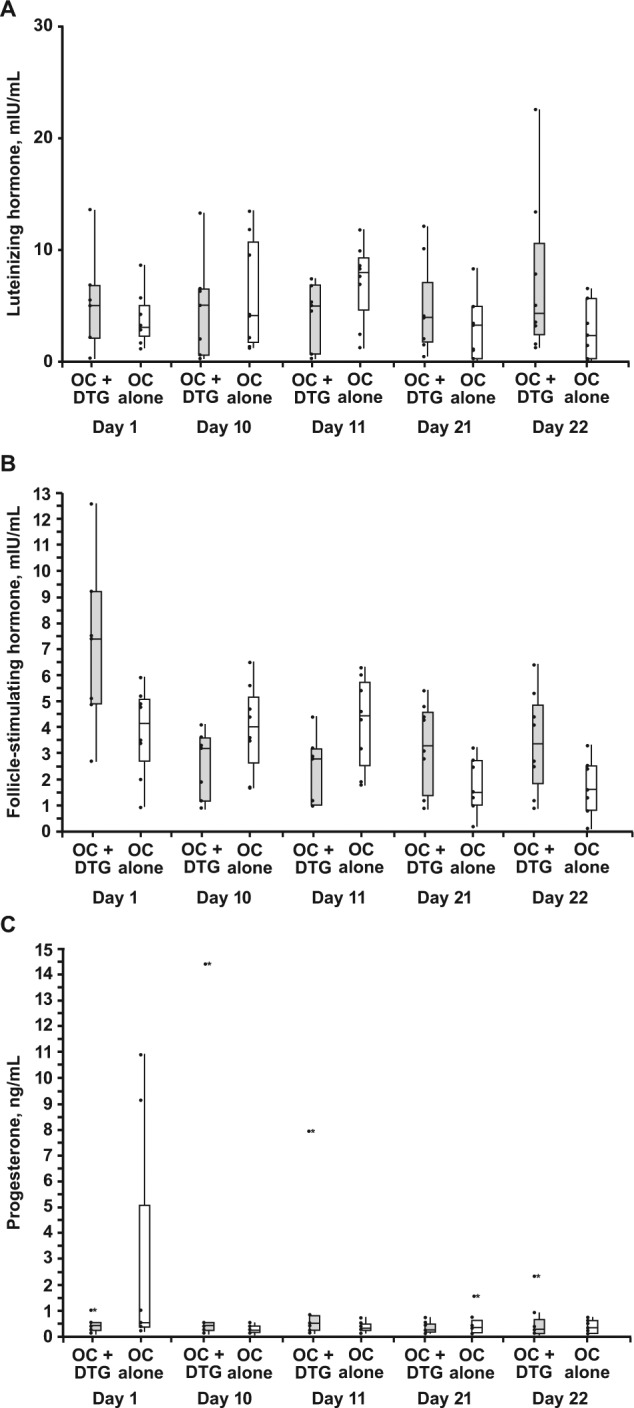
Predose (A) luteinizing hormone, (B) follicle-stimulating hormone, and (C) progesterone concentration by day and treatment. Abbreviations: DTG, dolutegravir; OC, organic cation.

Summaries of the concentration of individual biomarkers associated with fertility are shown in Figure 3. Overlap in LH, FSH, and progesterone concentrations was observed between those receiving DTG and placebo. There were no clinically meaningful differences in these markers between treatments.

### Safety

There were no SAEs or grade 3/4 AEs during the course of the study. No participant withdrew from the study because of AEs. The only drug-related AEs were headaches (n = 3, 19%), which were similar in frequency between treatments. One headache was grade 2 (moderate in severity), whereas all other drug-related headache AEs were grade 1 (mild in severity). There were no clinically significant changes in vital signs or laboratory parameters. No participant became pregnant during the course of the study.

## Discussion

The results of this study demonstrated that the PK of NGMN and EE were equivalent with and without coadministration of DTG. Furthermore, consistent with these findings, there was also no systematic trend or significant effect on the concentration of PD markers, including LH, FSH, and progesterone. Together, these results provide in vivo support for the coadministration of NGM/EE and DTG without dose adjustment.

DTG and NGM/EE coadministration was well tolerated, with only headache being reported as a drug-related AE in more than 1 participant, and all AEs were grade 1 or 2 in severity. Few AEs were observed with the 50-mg twice-daily dose of DTG used in this study. Although 50 mg once daily is the most commonly used dose in phase III trials, the 50-mg twice-daily dose is approved in the United States in those resistant to integrase strand transfer inhibitor^12^ and was selected for use in this study as the most likely scenario in which a potential drug interaction would be observed. The tolerability of NGM/EE and DTG 50 mg twice daily strongly suggests that the more commonly used 50-mg once-daily dose in HIV-1–infected individuals will also be well tolerated in combination with oral contraceptives.

The exposure of DTG with the 50-mg twice-daily dose reported in this trial is higher than in previous studies that evaluated this dose.^4,13^ The increase is a result of administration of DTG with food in this study, which has been shown to increase AUC by 33% to 66%, depending on the fat content of the meal.^14^ DTG can be given without regard to meals in HIV-infected patients.

There were limitations to study design and data extrapolation that should be addressed. The traditional study design for evaluating the effect of a drug on an oral contraceptive measures concentration of the estrogen and progestin components during a 28-day period with the oral contraceptive alone and then again in a second 28-day cycle combined with the investigational drug. This study used a nontraditional study design that implemented a 2-period crossover design within 1 menstrual cycle. The advantages of this design include a shorter study duration, which minimizes the number of individuals who may drop out and also limits cycle-to-cycle variability while providing a more stringent evaluation of safety and tolerability. However, the variation in levels of endogenous sex hormones and the expression of drug-metabolizing enzymes within a single menstrual cycle is a limitation of the 1-cycle design and must be taken into consideration. Therefore, in this study, a placebo-controlled crossover design was implemented to minimize this limitation of the 1-cycle design. Previous data suggest that oral contraceptive concentrations will not demonstrate wide variability within a cycle.^15-17^ Therefore, we also evaluated whether there was a period effect—that is, whether DTG administration in the first part of the cycle had a different effect on NGMN and EE compared with administration in the latter part of the cycle. The results of this analysis demonstrated that the order in which DTG or placebo treatment was given had no effect on concentrations of NGMN and EE.

NGM/EE is a combination estrogen-progestin oral contraceptive.^5^ Although it was selected in this study for its widespread use and fixed dose throughout the cycle, there are a number of similar products. Most combination oral contraceptives use EE for the estrogen component, whereas the progestin component may include norethisterone, levonorgestrel, gestodene, desogestrel, or NGM.^18^ Although there are some differences in metabolism among oral contraceptives, most oral contraceptives are primarily metabolized by CYP isozymes, including CYP3A4, as well as through sulfate and glucuronide conjugation in the liver and gut.^9^ The lack of effect of DTG on both the estrogen and progestin components in this study and on CYPs and UGTs based on in vitro data supports its use with caution with other brands of oral contraceptives.

In conclusion, the results of this study demonstrate that DTG did not affect the PK or PD of combination estrogen and progestin oral contraceptives, indicating that contraceptive efficacy should be maintained during coadministration of these drugs. NGM/EE can be administered with DTG without dose adjustment.
